# Exosomes: biogenesis, biologic function and clinical potential

**DOI:** 10.1186/s13578-019-0282-2

**Published:** 2019-02-15

**Authors:** Yuan Zhang, Yunfeng Liu, Haiying Liu, Wai Ho Tang

**Affiliations:** 1 Institute of Pediatrics, Guangzhou Women and Children’s Medical Center, Guangzhou Medical University, Guangzhou, 510623 Guangdong China; 2 Clinical Laboratory Department, Guangzhou Women and Children’s Medical Center, Guangzhou Medical University, Guangzhou, 510623 Guangdong China

**Keywords:** Exosome, Biogenesis, Biomarker, Therapeutic vehicle

## Abstract

Exosomes are nano-sized biovesicles released into surrounding body fluids upon fusion of multivesicular bodies and the plasma membrane. They were shown to carry cell-specific cargos of proteins, lipids, and genetic materials, and can be selectively taken up by neighboring or distant cells far from their release, reprogramming the recipient cells upon their bioactive compounds. Therefore, the regulated formation of exosomes, specific makeup of their cargo, cell-targeting specificity are of immense biological interest considering extremely high potential of exosomes as non-invasive diagnostic biomarkers, as well as therapeutic nanocarriers. In present review, we outline and discuss recent progress in the elucidation of the regulatory mechanisms of exosome biogenesis, the molecular composition of exosomes, and technologies used in exosome research. Furthermore, we focus on the potential use of exosomes as valuable diagnostic and prognostic biomarkers for their cell-lineage and state-specific contents, and possibilities as therapeutic vehicles for drug and gene delivery. Exosome research is now in its infancy, in-depth understanding of subcellular components and mechanisms involved in exosome formation and specific cell-targeting will bring light on their physiological activities.

## Introduction

Exosomes are small endosomal derived membrane microvesicles that have observed increasing attentions over the past decade. The presence of exosomes in extracellular space was identified as early as in late 1980s [1]. However, exosomes secreted from cells were initially proposed as cellular waste resulting from cell damage, or by-products of cell homeostasis, and have no significant impact on neighboring cells. It is only recently that these extracellular vesicles are functional vehicles that carry a complex cargo of proteins [2], lipids [3], and nucleic aids [2, 4, 5], be capable of delivering these cargos to the target cells they encounter, which may ultimately reprogram the recipient cells distal from their release. Thus, exosomes represent a novel mode of intercellular communication, which may play a major role in many cellular processes, such as immune response [6], signal transduction [7], antigen presentation [8]. As exosomes can be released by practically all eukaryotic cells, it is well considered that their cargos may greatly differ from each other for function of the originated cell types and their current state (e.g. transformed, differentiated, stimulated, and stressed). Thus, exosomes and their biologically active cargos may offer prognostic information in a range of diseases, such as chronic inflammation [9], cardiovascular and renal diseases [10, 11], neurodegenerative diseases [12], lipid metabolic diseases [13] and tumors [14].

In this review, we endeavor to provide a brief description of exosome biogenesis, molecular properties, and exosomal functional activities in cell–cell communications, as established to date. In addition, strategies for their isolation and characterization also be summarized. Finally, we discuss the feasibility of exosomes as clinical biomarkers and therapeutic potential of engineered exosomes as vehicles for targeted therapy.

## Exosome biogenesis

Exosomes are constitutively generated from late endosomes, which are formed by inward budding of the limited multivesicular body (MVB) membrane. Invagination of late endosomal membranes results in the formation of intraluminal vesicles (ILVs) within large MVBs [15]. During this process, certain proteins are incorporated into the invaginating membrane, while the cytosolic components are engulfed and enclosed within the ILVs. Most ILVs are released into the extracellular space upon fusion with the plasma membrane, which are referred to as “exosomes” [16, 17]. Alternatively, these components are trafficked to lysosomes for degradation. Canonical exosomes display a particular biconcave or cup-like shape when produced by artificially drying during preparation, while they appear spheroid in solution under transmission electron microscopy [18]. Typically, they have a density range from 1.13 g/mL (B cell-derived exosomes) [19] up to 1.19 g/mL (epithelial cell-derived exosomes) [20] on sucrose gradients.

Evidence has revealed that the formation of ILVs requires the endosomal sorting complex required for transport (ESCRT) function. It is an intricate protein machinery composed of four separate protein ESCRTs (0 through III) that work cooperatively to facilitate MVB formation, vesicle budding, and protein cargo sorting [21, 22]. The ESCRT mechanism is initiated by recognition and sequestration of ubiquitinated proteins to specific domains of the endosomal membrane via ubiquitin-binding subunits of ESCRT-0. After interaction with the ESCRT-I and -II complexes, the total complex will then combine with ESCRT-III, a protein complex that is involved in promoting the budding processes. Finally, following cleaving the buds to form ILVs, the ESCRT-III complex separates from the MVB membrane with energy supplied by the sorting protein Vps4 [21]. Despite the controversy of whether exosome release is an ESCRT-regulated mechanism, different ESCRT components and ubiquitinated proteins have already been identified in exosomes isolated from various cell types. Additionally, the typical exosomal protein Alix, which is associated with several ESCRT (TSG101 and CHMP4) proteins, has been reported to participate in endosomal membrane budding and abscission, as well as exosomal cargo selection via interaction with syndecan [23]. These observations led to a hypothesis implicating ESCRT function in exosomal biogenesis.

Interestingly, recent evidence favors an alternative pathway for sorting exosomal cargo into MVBs in an ESCRT-independent manner, which seems to depend on raft-based microdomains for the lateral segregation of cargo within the endosomal membrane. These microdomains are thought to be highly enriched in sphingomyelinases, from which ceramides can be formed by hydrolytic removal of the phosphocholine moiety [24]. Ceramides are known to induce lateral phase separation and coalescence of microdomains in model membranes. Moreover, the cone-shaped structure of ceramide might cause spontaneous negative curvature of the endosomal membrane, thereby promoting domain-induced budding. Consequently, this ceramide-dependent mechanism emphasizes a key role of exosomal lipids in exosome biogenesis [25]. Proteins, such as tetraspanins, also participate in exosome biogenesis and protein loading. Tetraspanin-enriched microdomains (TEMs) are ubiquitous specialized membrane platforms for compartmentalization of receptors and signaling proteins in the plasma membrane [26]. It has been shown that TEMs together with tetraspanin CD81 play a key role in sorting target receptors and intracellular components toward exosomes [27]. Apparently, several specialized mechanisms exist to ensure the specific sorting of bioactive molecules into exosomes, either the ESCRT-dependent or -independent mechanism (involved tetraspanins and lipids), may act variously depending on the origin of the cell type.

In addition to exosomes, other types of membrane vesicles produced by cells include plasma membrane-budded microvesicles (MVs) and apoptotic bodies. MVs are heterogeneous populations of membrane vesicles generated by outward budding from the plasma membrane. They are 100–1000 nm in size with variable shapes, and are predominately characterized as products of platelets, endothelial cells (ECs), and red blood cells. The density of MVs has been reported to be between 1.25 and 1.30 g/mL [28]. Apoptotic bodies are exclusively released from the plasma membrane during the late stage of apoptosis, range in size from 1 to 5 mm, comparable to that of platelets, and contain several intracellular fragments, cellular organelles, membranes, and cytosolic contents. Apoptotic bodies are closed structures with a higher sucrose gradient density than MVs, ranging from 1.18 to 1.28 g/mL [29]. The features and characteristics of these cell-derived MV types are listed in Table 1. Finally, the comparatively smaller size and unified shape allow exosomes to successfully escape clearance by the mononuclear phagocyte system, not only prolonging their circulation time, but also implying their superiority in cell–cell communication.

**Table 1:** Characteristics of main extracellular vesicles

Feature	Exosome	Apoptotic body	MV
Size	Homologous 30–100 nm	Heterogeneous 1–5 μm	Heterogeneous 100–1000 nm
Markers	Membrane impermeable (PI negative) CD63, TSG101, Alix, flottilin	Membrane permeable (PI positive) Annexin V, DNA, histones	Membrane impermeable (PI negative) integrin, selectin, flotillin-2
Density	1.13–1.19 g/mL	1.16–1.28 g/mL	1.25–1.30 g/mL
Contents	Protein, lipid, different RNA species, and DNA	Cytosolic content (protein, RNAs, fragmented DNA) and cellular organelles	Protein, lipid, different RNA species, and DNA
Determinant of controlled contents	The cellular origin and physiological state of the cell	The cellular origin and stimuli	No direct correlation
Lipids	A major sorting of lipidic molecules from the parental cells (include BMP)	Characterized by phosphatidylserine externalization	The lipid contents are primarily derived from plasma membrane, and resemble the parental cells (without BMP)
Origin	Multivesicular bodies fusion with plasmatic membrane	Cellular debris, plasma membrane blebbing during cell apoptosis	Direct outward budding or blebbing from the plasma membrane
Mechanism of release	Constitutive or inducible, depending on the cell type of origin	Rho-associated kinase I and myosin ATPase activity	Relocation of phospholipids to the outer membrane, cytoskeleton rearrangements, generation of membrane curvature, and vesicle release
Detection methods	Electron microscopy, Western blot for exosome enriched markers	Flow cytometry, electron microscopy,	Flow cytometry, electron microscopy
Isolation methods	Ultracentrifugation (100,000–200,000×*g*) filtration, density gradient Immunoprecipitation, Immune affinity capture and ExoQuick precipitation methods	Ultracentrifugation (10,000–20,000×*g*)	No standardized methods
Size determination and quantification	Dynamic light scattering Nanoparticle tracking analysis Surface plasmon resonance		
References	[19, 20, 48, 50, 122–124]	[29, 75, 125, 126]	[28, 48, 123, 124, 127, 128]

*MV* Microvesicle, *BMP* bone morphogenetic protein, *PI* propidium iodide

## The complex architecture of exosomes

Exosomes have been regarded as mini version of the parental cell, for the complex architecture of exosomes in terms of specially sorted proteins, lipids, nucleic acids, and respective content that highly dependent on the status quo of the cell type of origin. A large variety of constitutive elements have been identified in exosomes from different cell types, including approximately 4400 proteins, 194 lipids, 1639 mRNAs, and 764 miRNAs, which illustrate their complexity and potential functional diversity [30, 31]. Typically, exosomes are highly enriched in proteins with various functions, such as tetraspanins (CD9, CD63, CD81, CD82), which take part in cell penetration, invasion, and fusion events; heat shock proteins (HSP70, HSP90), as part of the stress response that are involved in antigen binding and presentation; MVB formation proteins that are involved in exosome release (Alix, TSG101); as well as proteins responsible for membrane transport and fusion (annexins and Rab) [32]. Among these proteins, certain members participate in exosome biogenesis (Alix, flotillin, and TSG101), rendering exosomes distinct from the ectosomes released upon plasma membrane shedding, while others specifically enriched in exosomes are widely used as exosomal marker proteins (e.g. TSG101, HSP70, CD81, and CD63). A detailed summary of protein components found in exosomes is shown in Table 2.

**Table 2:** Common protein components of exosomes

Protein category and description	Examples
Tetraspanins	CD9, CD63, CD81, CD82, CD37, CD53
Heat shock proteins (HSP)	HSP90, HSP70, HSP27, HSP60
Cell adhesion	Integrins, Lactadherin, Intercellular Adhesion Molecule 1
Antigen presentation	Human leukocyte antigen class I and II/peptide complexes
Multivesicular body Biogenesis	Tsg101, Alix, Vps, Rab proteins
Membrane transport	Lysosomal-associated membrane protein 1/2, CD13, PG regulatory-like protein
Signaling proteins	GTPase HRas, Ras-related protein, furloss, extracellular signal-regulated kinase, Src homology 2 domain phosphatase, GDP dissociation inhibitor, Syntenin-1, 14-3-3 Proteins, Transforming protein RhoA
Cytoskeleton components	Actins, Cofilin-1, Moesin, Myosin, Tubulins, Erzin, Radixin, Vimentin
Transcription and protein synthesis	Histone1, 2, 3, Ribosomal proteins, Ubiquitin, major vault protein, Complement factor 3
Metabolic enzymes	Fatty acid synthase Glyceraldehyde-3-phosphate dehydrogenase Phosphoglycerate kinase 1 Phosphoglycerate mutase 1 Pyruvate kinase isozymes M1/M2 ATP citrate lyase ATPase Glucose-6-phosphate isomerase Peroxiredoxin 1 Aspartate aminotransferase Aldehyde reductase
Trafficking and membrane fusion	Ras-related protein 5, 7 Annexins I, II, IV, V, VI Synaptosomal-associated protein Dynamin, Syntaxin-3
Antiapoptosis	Alix, Thioredoxine, Peroxidase
Growth factors and cytokine	Tumor Necrosis Factor (TNF)-α, TNF Receptors, Transforming growth factor-β
Death receptors	FasL, TNF-related apoptosis inducing ligand
Iron transport	Transferrin receptor
References	[18, 99, 129, 130]

Aside from selected proteins, exosomes also contain different patterns of RNAs that can be incorporated into recipient cells. RNA sequencing analysis showed that microRNAs (miRs) were the most abundant in human plasma derived exosomal RNA species, making up over 42.32% of all raw reads and 76.20% of all mappable reads [33]. Other RNA species including ribosomal RNA (9.16% of all mappable counts), long non-coding RNA (3.36%), piwi-interacting RNA (1.31%), transfer RNA (1.24%), small nuclear RNA (0.18%), and small nucleolar RNA (0.01%). Once miRs are packed into exosomes, they can undergo unidirectional transfer between cells, resulting in the establishment of an intercellular trafficking network, which, in turn, elicits transient or persistent phenotypic changes of recipient cells [8]. MiRs, such as miR-214, miR-29a, miR-1, miR-126, and miR-320, which participate in angiogenesis, hematopoiesis, exocytosis, and tumorigenesis, have already been reported in exosome-based cell to cell communication [34]. Interestingly, besides miRs, long RNA species, especially long non-coding RNAs (lncRNAs) and circular RNAs (circRNAs) have recently been reported to be existed in exosomes, and impact a variety of biological processes including the development of cancer [35]. They may function together to transduct cell signals so that local cellular microenvironments will be altered or maintained. Early in 2013, Kogure and his group identified several dramatically altered lncRNAs in human hepatocellular cancer (HCC) cell-derived exosomes [36]. Among them, the novel lncRNA TUC339 was the most highly significantly expressed one, which was functionally implicated in modulating tumor cell growth and adhesion. Thus, they prompted that exosomes-mediated transfer of intercellular functionally active lncRNA as a mechanism of intercellular signaling in HCC. Later, Alice et al. reported that lncRNA H19 could be packaged inside CD90^+^Huh7 cells-derived exosomes, and be delivered to endothelial cells (ECs), influencing ECs in a pro-metastatic way via the exosome-mediated vascular endothelial growth factor (VEGF) increase [37]. These studies indicate that exosome-mediated transfer of lncRNA is an important mechanism existed in tumor development, and play a crucial role in regulation of the tumor microenvironment via influencing major cellular pathways. Other lncRNAs transmitted by exosomes include lncRNA CRNDE-h in colorectal cancer [38], lncARSR in sunitinib resistance of renal cancer [39], lncRNA Hotair in rheumatoid arthritis [40], lincRNA-p21 and ncRNA-CCND1 in bleomycin-induced DNA damage [41]. CircRNAs were demonstrated high stability, and were not susceptible to exonuclease cleavage, which proposed a possible tumor diagnostic marker. In 2015, Li et al. identified existence of circRNAs in exosomes through RNA sequencing analyses of hepatic MHCC-LM3 cancer cells and cell-derived exosomes [42]. In a comparison of healthy donors and colorectal cancer patients, 67 circRNAs were missing and 257 new circRNA species were detected in cancer patients. Further overexpression of miR-7 in HEK293T cells and MCF-7 cells showed significant downregulation of circRNA CDR1as in exosomes, suggesting that the process of circRNAs entering exosomes may be regulated by intracellular miRs. In a recent study, Exosomal circRNA_100284 derived from arsenite-transformed cells, has been reported to promote malignant transformation of human hepatic cells, via regulation of EZH2 by miR-217 [43].

The bioactivity of exosomes exists not only in their proteins and nucleic acids, but also in their lipid components. Generally, exosomes are enriched in phosphatidylserine (PS), phosphatidic acid, cholesterol, sphingomyelin (SM), arachidonic acid and other fatty acids, prostaglandins, and leukotrienes, which account for their stability and structural rigidity (listed in Table 3). Moreover, exosomes also have some functional lipolytic enzymes, which can produce units of various bioactive lipids autonomously. These exosomal bioactive lipids may be internalized into recipient cells, concentrating lipid mediators within the endosomes. Evidence has revealed that accumulation of prostaglandins and fatty acids brought by exosomes during 1 h can result in micromolar concentrations for prostaglandins, and millimolar concentrations for fatty acids, which are enough to trigger prostaglandin-dependent biological responses [44]. Meanwhile, exosomal lipids may interact with lipid transfer proteins in recipient cytosolic, such as fatty acid binding proteins (FABPs), or receptors such as PPARg for the 15dPGJ2, for conformation of the cytosolic complex AA/FABP/PPARg [45], which will be further addressed to the nucleus. As a result, the exosome derived 15d-PGJ2 and PGE2 may provide a natural way to supply intracellular PGE2. The endosomal AA brought by exosomes can be delivered to the PGE synthase and COXs present in endoplasmic reticulum for an additional PGE2 synthesis. Similarly, the unsaturated 22:6 fatty acid DHA released from exosomes can potentially be addressed to the microsomal antioestrogen binding site in the recipient endoplasmic reticulum [46] and inhibit the activity of the cholesterol epoxide hydrolase which cleaves the cholesterol 5,6 epoxide into cholestanetriol [47]. Since exosomes can be released and taken up by target cells to modulate cell lipid metabolism, exosome-mediated intercellular lipid exchange should be taken into account in the pathogenesis of cholesterol-related storage disease, such as atherosclerosis. Internalization of T cell-derived exosomes by monocytes via the PS receptor has been shown to effectively facilitate cholesterol accumulation into lipid droplets, implying a role of exosomes in atherosclerosis development [20]. Since exosomes have been shown to play a role in lipid-related pathologies, the lipid content of exosomes may act as another molecular signature for disease diagnosis and prognosis, in addition to protein and RNA biomarkers.

**Table 3:** Lipid-related enzymes and bioactive lipids in exosomes

lipid category and description	Lipid related enzymes	Functional effects
LTA4, LTB4, LTC4	LTA4 hydrolase, LTC4 synthase	Triggering polymorphonuclear [131] leukocyte migration
PGE2, 15d-PGJ2	COX-1, COX-2	Immunosuppression, [44] PPARγ ligand
PGE2	PGE synthase	Inflammation [4]
PA	PLD2, DGK	Increasing exosome production [132, 133]
AA, LPC	cPLA2, iPLA2	Accounting for the membrane curvature [44]
/	sPLA2 IIA, sPLA2 V	Prostaglandin biosynthesis [44]
Ceramides	nSMase2	Sorting cargo into MVBs [134]
Cholesterol	/	Regulating exosome secretion [135]
BMP	/	MVB formation [135] and subsequent ILV biogenesis [136]
PS	/	Being involved in exosome fate [13, 122]
SM	/	Triggering calcium influx [135]

*LA4, LTB4, LTC4* Leukotriene; *COX-1, COX-2* cyclooxygenases; *PGE2, 15d-PGJ2* prostaglandins; *PLD2* phospholipase D2; *DGK* diglyceride kinase; *PA* phosphatidic acid; *PLA2* phospholipases A2; *cPLA2* calcium-dependent phospholipases A2; *iPLA2* calcium-independent phospholipases A2; *AA* arachidonic acid; *LPC* lysophosphatidylcholine; *sPLA2 IIA; V* secreted phospholipases A2 IIA and V; *nSMase2* neutral sphingomyelinase 2; *BMP* Bis(monoacylglycero)phosphate, also called LBPA; *PS* phosphatidylserine; *SM* sphingomyelin

## Exosome-mediated intercellular communication

**Fig. 1:**
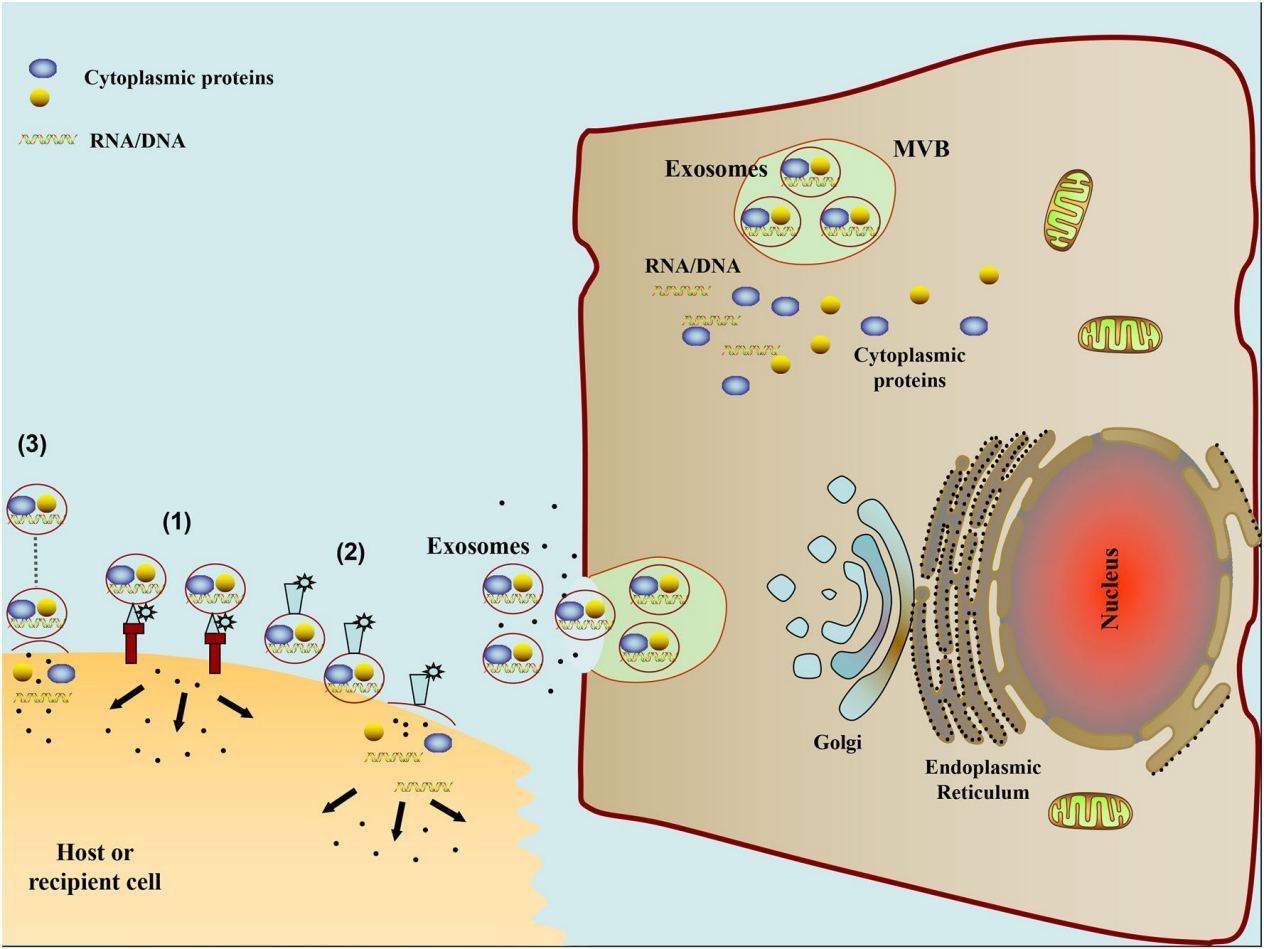
The schematic diagram of pathways involved in exosome mediated cell-to-cell communication. (1) Exosomes signal recipient cells via direct surface-bound ligands. (2) Exosomes transfer activated receptors to recipient cells. (3) Exosomes may epigenetically reprogram recipient cells via delivery of functional proteins, lipids, and RNAs

Traditionally, cells communicate with neighboring cells through direct cell–cell contact including gap junctions, cell surface protein/protein interactions, while communicating with distant cells through secreted soluble factors, such as hormones and cytokines, to facilitate signal propagation [48]. Moreover, electrical and chemical signals (e.g. nucleotides, lipids, and short peptides) are also involved for communication [49]. Interestingly, it is now recognized that exosomes with a cell-specific cargo of proteins, lipids, and nucleic acids may act as a novel intercellular communication mechanism. This concept is based on the observation that exosomes released from parental cells may interact with target cells, leading to the subsequent influence of target cell behavior and phenotype features [50]. The success of exosomal biological applications is highly dependent on effective delivery of genetic materials, which can be achieved via receptor-ligand interactions, direct fusion of membranes, or internalization via endocytosis [51]. Once internalized, exosomes may fuse with the limiting membrane of endosomes, resulting in the horizontal genetic transfer of their content to the cytoplasm of target cells. The bioactive molecules contained in exosomes have been shown to impact target cells via the following mechanisms: (1) direct stimulation of target cells via surface-bound ligands; (2) transfer of activated receptors to recipient cells; and (3) epigenetic reprogramming of recipient cells via delivery of functional proteins, lipids, and RNAs [52] (Fig. 1). As a result, parental cells can communicate with specific proximal or distal target cells through exosome amplification.

In immune system, exosomes have an important function in immunoregulation, including antigen presentation, immune activation, immune suppression, and immune tolerance via exosome-mediated intercellular communication. Exosomes derived from CD4^+^ T cells and CD8^+^ T cells can bind to dendritic cells (DCs) through peptide/major histocompatibility complex MHC/TCR and ICAM-1/LFA-1 interactions, which lead to the apoptosis of DCs and thus mediate the DC-mediated T cell silence in antigen-specific way [53]. Exosomes secreted by regulatory T cells contain Let-7b, Let-7d, and microRNA-155, which are able to inhibit Th1 immune response and mediate immune suppression [54]. In addition, CD73-expressing Treg-derived exosomes can produce adenosine which may further inhibit the activation and proliferation of CD4^+^ T cells [55, 56]. Meanwhile, B lymphoblast-derived exosomes have also been shown to induce human and mouse-antigen specific T cell activation, for the presence of MHC–peptide complexes, and even co-stimulatory molecules on them [57]. DCs are professional antigen presenting cells with the unique capacity to induce primary and secondary immune responses. It has been reported that exosomes derived from DCs pulsed with tumor peptide can eradicate or suppress growth of established murine tumors via presentation of class II-peptide complexes to naive T lymphocytes and the priming of specific cytotoxic T lymphocytes in vivo [58].

Mesenchymal stem cells (MSCs) derived exosomes are prompted to be the main effects on wound healing. The therapeutic capacity of MSC-‘exosomes’ derived from different organs, have been tested in various disease models, demonstrating a similar or even superior functional capacity to MSCs themselves, such as reducing myocardial infarction size [59, 60], preventing adverse remodeling after myocardial ischemia/reperfusion injury [61], providing therapeutic effects in cutaneous wound healing [62], acute kidney injury [63], hepatic injury [64, 65], neonatal lung injury [66], promoting survival of retinal ganglion cells in optical nerve crush [67], ameliorating retinal laser injury [68] and orchestrating neurological protection by the transfer of miRs [69].

Surprisingly, in contrast with wide spread cell origin, exosomes do not randomly interact with any recipient cell that happens to be in the vicinity. They may display distinct tissue/cell homing, probably on account of their high expression levels of adhesion molecules, such as integrins and tetraspanins, and their potential target capability [70]. Hence, the selective transmission of exosomal genetic information makes them attractive candidates for the diagnosis and treatment of diseases.

## Methods for exosome purification

Typically, exosomes are characterized by size, morphology, flotation density, and the presence of marker proteins, such as Alix, TSG101, flotillin 1, HSP70, and CD9. Different techniques for isolation of exosomes have been suggested. Generally, exosomes can be isolated from conditioned cell culture media or bodily fluids by differential centrifugation [71], size exclusion chromatography [72], immune affinity capture [73], commercially available kits, or microfluidic technologies. Each approach has its advantages and disadvantages, and may be dictated by the sample source and intended use of exosomes.

Current dogma states that ultracentrifugation at high speeds is the best method to purify exosomes. An elegant study by Webber has shown that using an ultracentrifuge method on a sucrose cushion was able to obtain a highly pure exosomal fraction [74]. However, this was confirmed only valid for exosomes purified from cell culture media, but not the urine and serum with complex mixture of many components, for possible co-sedimentation of protein aggregates and non-specifically bound proteins. Filtration through a series of filters down to 100 nm pore size followed by centrifugation is an efficient way to collect exosomes away from smaller protein contaminants, although yielding relatively high protein content, still risks impurity because of the fragmentation of larger microparticles into smaller vesicles under filtration pressure [75]. An immunoaffinity pull-down method with the use of antibody-loaded magnetic cell beads can also be performed to selectively enrich exosomes, which is regarded as having the advantage of specificity, but yields are often low [76]. As a result, the most generally accepted and widely used method for purifying exosomes from culture media is to use a well-defined series of serial centrifugation steps that remove cells and microvesicles, followed by concentration via ultracentrifugation and subsequent density gradient purification. However, it is time-consuming, labor-intensive, and in need with expensive equipments. In recent years, several commercially available kits have been emerged to enrich exosomes, including ExoQuick (System Bioscience) [77], the Total Exosome Isolation kit (Invitrogen) [78], Exo-spin (Cell Guidance System) [79]. They are primarily size-based precipitation approaches with greatly shortening operation time less than 2 h, and have been proven to be efficient, reliable and reproducible when compared with other methods.

However, challenges exist not just in exosome isolation, but rather in rapid and accurate quantification of exosomes with minimal sample preparation, to allow detection of tiny pathological changes of disease. Finally, dynamic light scattering [80] and nanoparticle tracking analysis [81], the alternative methods relying on direct particle counting, have been more recently developed. Despite the rapid, reliable, semi-quantification capabilities of both approaches, a weakness of each is their inability to specifically identify exosomes, partly through particles of a given size. Notably, in recent years, surface plasmon resonance in combination with antibody microarrays specific to exosome membrane proteins of extracellular domains was utilized to quantitatively detect and characterize tumor-derived exosomes without enrichment or purification, which provides an easy, efficient, and novel method for the detection and monitoring of exosomes, thus creating an avenue to advance diagnostic applications of exosomes [82]. The advantage and disadvantage of various methods have been summarized in Table 4.

**Table 4:** Methods for exosome characterization

Isolation methods	Procedures	Advantages	Limitations
Ultracentrifugation [42]	400×*g* (to remove cells and large cell debris) 10,000–20,000×*g* (to remove large debris and intact organelles) 100,000–150,000×*g* (to pellet exosomes)	Golden standard, obtain highly pure exosomal fraction	1. Only valid for exosomes purified from cell conditioned medium, but not the body fluids with complex mixture of many components 2. It sediments exosomes as well as other vesicles, proteins, and/or protein-RNA aggregates 3. Time-consuming, labor-intensive, and requires expensive equipment
Size exclusion (filtration [75] or chromatography [137])	Filtration through a series of filters down to 100 nm pore size followed by centrifugation (100,000×*g*) to concentrate	Collect exosomes away from smaller protein contaminants	Risk of impurity or fragmentation of larger vesicles under filtration pressure
	CCM or biofluid is dissolved in the mobile phase followed by passing through the stationary phase, wherein the various constituents of the mixture travel at different speeds so as to separate	Preserves the integrity and biological activity of exosomes	1. Deformation and breaking-up of larger vesicles, which may potentially skew results 2. Requiring a long run time, limiting its scalability for high-throughput applications
Immune affinity capture [138]	Incubate CCM with specific microbeads to bind exosomes, separate exosome-bound microbeads from CCM using solid support magnet or flow cytometry	Collect exosomes with specificity	Yields are often quite low
ExoQuick precipitation methods [139]	This precipitation solution is combined with biofluid containing exosomes and is incubated overnight at 4 °C. The mixture is then centrifuged at low speed to form a pellet containing exosomes	1. Enable high-throughput, quantitative isolation of exosomes from low sample volumes 2. No need specialized equipment and shorten the operation time just in less than 2 h 3. Be efficient, reliable and reproducible	Co-precipitating non-vesicular contaminants, such as lipoproteins and polymer materials
Microfluidic technologies (ExoChip) [140]	Immunoaffinity, sieving, and trapping exosomes on porous structures	Quantitative and high-throughput analysis of exosome contents with high sensitivity	Inadequate quality control and normalization across study groups, not yet in clinical use

Identification and quantification methods	Mechanical principles	Advantages	Disadvantages
Dynamic light scattering [141]	Determine the fluctuations in the intensity of light scattered from nanoscale particles in solution, thereby calculating the sizes and the diffusion coefficients of the particles under Brownian motion	1. Accurate, reliable, and repeatable particle size analysis in 1 or 2 min 2. Size measurement of molecules with MW < 1000 Da 3. Low volume requirement (as little as 2 µL)	Low refractive index of vesicles and a bias towards detection of larger particles renders it problematic to distinguish MVs from exosomes in mixtures
Nanoparticle Tracking Analysis [81]	Quantification of particles in the range of 50–1000 nm in liquid suspension through real-time image of light scattered from individual nanoscale particles moving under Brownian motion	Detection of single vesicles with a diameter as low as 50 nm	1. Only semi-quantification of the exosomes/microvesicles preparations 2. Risk of no detection of nanoparticles with a certain size or optical density 3. Considerable intra-assay count variability exists
Surface plasmon resonance [142]	Sensitively detects molecular interactions occurring on the metal/dielectric interface through monitoring minute changes in the refractive index resulting from adsorption of molecules onto the metal surface, which is then converted into the surface bound mass and related to the concentration of exosomes in solution	1. Competence for the total mass of exosomes, including proteins, lipids, and nucleotides 2. Compatibility with selective surface immobilized capture molecules for the binding partner; under investigation 3. Small sample volumes	Inadequate quality control and normalization across study groups; not yet in clinical use

Of note, the lack of techniques to rapidly isolate, purify, quantitate, and identify exosomes are main drawbacks hampering the clinical use of exosomes. Although recently established technologies may be able to mitigate these drawbacks, additional challenges include the reproducibility and consistency of the product lots, and inadequate quality control and standardization across study groups. Therefore, much more researches are needed for robust validation of these methods to be successfully implemented in clinical diagnosis.

## Exosomes in diagnostics

Over the past few years, exosomes have been discovered in almost all body fluids, including blood, urine [83], saliva [84], breast milk [85], cerebrospinal fluid [86], semen [87], amniotic fluid [88], and ascites [89]. These exosomes with specific profile of miRs, proteins, and lipids can mirror the cellular origin and its physiological state, as a “fingerprint” or a “signature” of the donor cell. Therefore, exosomes and their cell- or condition-specific cargos may better reflect the cellular processes and be used as biomarkers of various diseases.

For example, serum level of exosomal miR-21 has been found to robustly distinguish patients with esophageal squamous cell cancer from patients who have benign diseases [90]. Moreover, it was also found to be significantly higher in patients with HCC than those with chronic hepatitis B or healthy volunteers as well [91]. Furthermore, Machida et al. investigated the diagnostic value of salivary exosomes, and found that their cargos of miR-1246 and miR-4644 were significantly higher in cancer patients with an increased area under the curve of 0.833, and have the potential for clinical diagnosis of pancreatobiliary tract cancer [92]. In an investigation of bovine milk with bacterial infection, Sun et al. compared miR expression profile of milk exosomes before and after intramammary infection, and found that exosomal bta-miR-142-5p, and -223 were significantly up-regulated in response to infection, thus provide a potential for early detection of bacterial infection of the mammary gland [93]. Moreover, exosomal RNAs from amniotic fluid can be used for CD24 Ala/Val SNP (rs52812045) genotyping, which is associated with faster progression of autoimmune diseases. Other than exosmal miR, the analysis of urinary exosome also provide protein biomarkers for kidney injury, such as Fetuin-A [94], aquapotin-1 [95], cyclic AMP-dependent transcription factor-3 [96], Wilms’ tumor-1 [97]. Urinary exosomes isolated from prostate cancer patients were even found to contain cancer signatures, e.g. prostate specific antigen, prostate cancer gene 3 [98].

Although the biological fluids are relatively easy to be acquired, and are rich in exosomes, the actual use of exosomal contents (proteins or miRs) as biomarkers has not yet been put into effect in clinical practice. Undoubtedly, the emergence of exosome-based molecular diagnostic tests will herald a new chapter in clinical diagnosis.

## Exosomes as drug delivery vehicles

Exosomes are nanoscale membrane vesicles with the exceptional ability to target specific cells or tissues. They may mediate a horizontal transfer of genetic material via interaction of surface adhesion proteins, resulting in modified biological activities of recipient cells. Besides, exosomes can be obtained from patients’ tissues or body fluids with excellent host bio- distribution and biocompatibility, which allows for diminished clearance by the mononuclear phagocyte system [99]. Thus, the issue of immunogenicity can be circumvented, and incorporated therapeutic agents can be delivered without the rapid clearance and toxicity. In recent years, an increasing number of studies have revealed that, in addition to being used as therapeutic entities themselves, exosomes could be regarded as attractive biological vesicles for the efficient delivery of biological therapeutics across different biological barriers to target cells [100–103].

Generally, a variety of therapeutic material, such as short interfering-RNA (siRNA), antagomirs, recombinant proteins, and anti-inflammatory drugs, can be encapsulated for exosome-mediated delivery via several delivery approaches, including: (1) Isolation of exosomes from donor cells ex vivo, and incorporated therapeutic agents into exosomes; (2) Encapsulation of donor cells with the therapeutic agent, which can be sorted into exosomes while exosomal formation; (3) Transfection of donor cells with the drug-encoding DNA, which can be expressed and sorted into exosomes [104]. Each approach has its advantage and limitation (Table 5), primarily depending on the unique characteristics of exosomes, the specific type of therapeutic agent, and site of disease. These artificial exosomes then are capable of delivering their cargos across the blood–brain barrier and confer an active biological effect exactly on target cells.

**Table 5:** The advantage and disadvantage of each type of therapeutics

Therapeutic application of exosomes	Type I	Type II	Type III
Drugs have been reported to be loaded in exosomes	Lipophilic small molecules such as antioxidant, curcumin [143], anticancer agents, Doxorubicin [144, 145] and Paclitaxel (PTX) [103], and a model drug Rhodamine 123 [103], catalase [101], exogenous siRNA [146, 147]	PTX [100], Etoposide, Carboplatin, Irinotecan, Epirubicin, and Mitoxantrone [148], Dox, Gentamicin, 5-Fluorouracil, or Carboplatin [108], catalase [149]	OVAC1C2 fusion complementary DNA [150], pDNA for catalase [151], GDNF [149], adeno-associated virus capsids [152]
Disadvantages	Relatively low loading capacity for already numerous proteins and nucleic acids in them	The therapeutic protein maybe degraded in host cells	The drug is limited for its encoding DNA should be expressed and sorted into exosomes
	/	Or the therapeutic protein should be incorporated into a polymer based nanocontainer before the loading into parental cells	/
	/	The amount of drugs loaded into exosomes is difficult to estimate for the process of loading procedure	/
Advantages	Make the quantity, standardization and uniformity of exosomal drug formulations much easier	Targeting exosomes to the disease site specifically	Exosomes may contain the encoded therapeutic protein, as well as its genetic material (DNA and mRNA)
Common merits	Non-cytotoxic effects, a high drug carrying capacity, and a low immunogenic profile		

In one of the first reports, peripheral blood exosomes incorporated with exogenous siRNAs were used for efficient silencing of the target MAPK gene in monocytes and lymphocytes [105]. In another study, Didiot et al. showed that exosomes can be harnessed to deliver siRNAs targeting Huntingtin mRNA to mouse primary cortical neurons, resulting in statistically significant bilateral silencing of Huntingtin mRNA and protein, and consequent therapy of Huntington’s disease [106]. Similarly, Yang et al. reported that brain endothelial cell-derived exosomes could deliver VEGF-siRNA across the blood–brain barrier in zebrafish and decrease the fluorescence intensity of labeled cancer cells [107]. Although almost all types of cells can release exosomes, the quantities of exosomes are relatively low, and the purification procedure is difficult. To deal with this problem, Jang et al. produced bioinspired exosome-mimetic nanovesicles through the breakdown of monocytes or macrophages. Similar characteristics with exosomes were maintained, but 100-fold higher production yield can be observed [108]. Experiments in mice revealed that these chemotherapeutic-loaded nanovesicles are capable of trafficking to tumor tissue and reduce tumor growth without adverse effects.

MSCs are multipotent cells with anti-inflammatory and/or immunosuppressive properties, and emerged as therapeutic agents in many diseases. In recent years, increasing evidences have revealed that the regenerative potential of MSCs are primarily mediated via paracrine factors, especially extracellular vesicles (EVs), including exosomes [109, 110]. Thus, extensive researches are currently underway for the use of MSCs-derived EVs as effective cell-free therapy. Pascucci et al. showed that MSCs loaded with PTX exhibited strong anti-tumor activity via production of a significant amount of PTX-loaded exosomes. These exosomes could package and deliver active drugs to human pancreatic cells, inducing a dose-dependent inhibition of cell proliferation, as well as 50% tumor growth in vivo [100]. Moreover, Shimbo et al. reported that synthetic miR-143 introduced into MSCs could be released and enveloped into exosomes. These exosome-derived miR-143 was functional and caused significant reduction of osteosarcoma cells migration [111].

Another therapeutic avenue involves the genetic modification of donor cells to product exosomes contained the encoded therapeutic proteins, as well as DNA or mRNA. Exosomes released from genetically modified macrophages that were transfected with a plasmid DNA (pDNA) encoding a potent antioxidant enzyme, catalase, could result in successful gene therapy of Parkinson’s disease. These genetically modified macrophages derived exosomes were enveloped with catalase genetic material, including pDNA, mRNA, active catalase, and NF-κB, a transcription factor. They were capable of efficiently transferring their contents to contiguous neurons resulting in de novo protein synthesis in target cells. Notably, the transfected brain tissue showed month-long expression levels of catalase, and a substantial (over 40 days) and profound anti-inflammatory and neuroprotective effects in mice with neuroinflammation [101]. These studies cooperatively demonstrated that exosomes could be used as more efficient delivery vehicles to direct specific targeting of new therapeutics without immunogenicity and adverse effects.

In recent years, exosomes derived from dendritic cells (Dex) pulsed with tumor peptides were actively investigated as clinical cell-free cancer vaccines [112]. Dex was found to possess molecules necessary for antigen presentation, such as MHC class I, MHC class II, costimulatory adhesion molecules, each of which facilitates the functionality of Dex in vivo [113]. Evidences have revealed that Dex is capable of promoting tumor cell-specific cytolysis and eradicating growth of established murine tumors in a CD8^+^ T cell-dependent manner. Since 2005, the first Phase I clinical trial using autologous MAGE antigen-loaded Dex had been performed on 15 stage III/IV melanoma patients [114]. One partial response and some other tumor regressions were observed in the absence of toxicity. Later, a phase II clinical trial using the second generation of Dex, exosomes derived from interferon (IFN)-γ-maturated DCs (IFN-γ-Dex) pulsed with tumor-associated antigenic peptides, was carried out in patients bearing advanced non-small cell lung cancer [115]. Besse et al. found that IFN-γ-Dex increased NK cell functions and related antitumor immunity in NKp30-dependent manner, resulting in longer progression-free survival. In order to further improve the efficacy of Dex immunotherapy, engineered Dex was developed to carry tumor antigen-associated (TAA) proteins or mRNAs, thereby inducing CTL response, leading to inhibition of tumor growth [116, 117]. Meanwhile, engineered inhibition of some immune checkpoint molecules, such as CTLA-4 or PD-1, in combination with Dex may enhance antitumor T and B cell response, improving the outcomes of future clinical trials [118, 119].

As a new vaccine strategy for cancer immunotherapy, Dex remain have promising potential for improvements. Future research and clinical trials should focus on how to enhance particular immunostimulatory characteristics: such as greater surface expression of costimulatory molecules, lower expression of immunoregulatory molecules. The choice of TAAs might also be improved to increase the delivered quantity of TAAs to Dex, so as to enhance CTL response. Moreover, engineered Dex has TNF, FasL [120], and TRAIL on its surface may help direct trigger caspase activation and consequent apoptosis of tumor cells [121]. The successful engineered Dex will provide promising therapeutic opportunities for patients with serious cancers.

## Conclusions

In the past few years, an exponential increase studies focused on the biological characteristics of exosomes. The exosomal secretion from various types of cells, existence in almost all kinds of body fluids, and function as mediators of cell–cell communication, which make exosomes play a crucial role in both physiological and pathological processes. Moreover, the capacity of exosomes to envelope with a wide range of content including lipids, RNAs, and proteins to signal specific recipient cells or tissues, makes them a promising diagnostic biomarker and therapeutic tool for treatment of cancers and other pathologies. The clinical application of exosomes is still in its infancy, further investigations will contribute to find out cost- and time-effective nanotechnologies for large-scale production of exosomes. Indeed, exosomes have the capacity of carrying specific therapeutic drugs based on the treatment of corresponding diseases, it is necessary to identify appropriate strategies for further tailoring exosomes as drug delivery vesicles with high drug loading capacity, high specificity, non-cytotoxic effect and low immunogenicity. Furthermore, the isolation, classification, and purification of exosomes need to be standardized to make sure the clinical application of exosomes feasible. Undoubtedly, exosomes represent a promising tool in the field of nanomedicine and may provide the solution to a variety of medical mysteries faced today.

## Abbreviations

MVB: multivesicular body
ILV: intraluminal vesicle
MV: microvesicle
EC: endothelial cells
HSP: heat shock protein
miR: microRNA
lncRNAs: long non-coding RNA
circRNA: circular RNAs
HCC: hepatocellular cancer
PS: phosphatidylserine
PA: phosphatidic acid
FABPs: fatty acid binding proteins
VEGF: vascular endothelial growth factor
SM: sphingomyelin
ESCRT: the endosomal sorting complex required for transport
TEM: tetraspanin-enriched microdomains
DCs: dendritic cells
UC: ultracentrifugation
LA: lung adenocarcinoma
ESCC: esophageal squamous cell cancer
CKD: chronic kidney disease
siRNA: short interfering-RNA
MSCs: mesenchymal stem cells
EVs: extracellular vesicles
pDNA: plasmid DNA
Dex: exosomes derived from dendritic cells
TAA: tumor antigen associated
